# N-acetylcysteine as adjuvant therapy for hospitalized Covid-19 patients: A single-center prospective cohort study 

**DOI:** 10.22088/cjim.14.3.553

**Published:** 2023

**Authors:** Siamak Afaghi, Negin Moghimi, Nasser Malekpour Alamdari, Fatemeh Sadat Rahimi, Rana Irilouzadian, Farzad Esmaeili Tarki, Morvarid Moghimi, Sara Besharat, Hossein Salehi Omran, Anita Karimi

**Affiliations:** 1 Research Institute of Internal Medicine, Shahid Modarres Hospital, Shahid Beheshti University of Medical Sciences, Tehran, Iran; 2 Clinical Research and Development Center, Department of Surgery, Shahid Modarres Hospital, Shahid Beheshti University of Medical Sciences, Tehran, Iran; 3 School of Chemical Engineering, Faculty of Engineering, University of Tehran, Tehran, Iran; 4 Department of Radiology, Shahid Labafi Nejad Hospital, Shahid Beheshti University of Medical Sciences, Tehran, Iran; *: Co-first authors co-equally contributed to this study.

**Keywords:** N-acetylcysteine, Covid-19, Hospitalization

## Abstract

**Background:**

Whilst over two years have passed since the COVID-19 pandemic's emergence, the proper management of the disease remains challenging. N-acetylcysteine (NAC) as a potentially effective therapeutic option has been suggested by studies, while the exact clinical role of this agent is yet to be evaluated.

**Methods:**

This prospective case-control study was conducted in a major referral respiratory center in Tehran, Iran. We enrolled 217 patients treated with an intravenous daily dose of 1500 mg NAC as a case group; and 245 control patients who did not receive NAC. Two groups were matched based on other treatments, socio-demographics, medical history, and comorbidities.

**Results:**

After ten days of adjuvant therapy with NAC, patients in the NAC group and control group had median room-air SpO2 of 91% and 88%, respectively (P=0.02). Also, the SpO2 to FiO2 ratio had a median of 463 and 421 in the case and control groups, respectively (P=0.01). Furthermore, the case group's hospitalization period was three days shorter (P=0.002). Further, cough, dyspnea, and decreased appetite were reported to have a significantly lower incidence in the case group (P=0.03, 0.001, 0.008).

**Conclusion:**

We showed that a daily intravenous dose of NAC in hospitalized COVID-19 patients could shorten the hospital stay and improve some clinical symptoms; however, it does not remarkably improve the risk of ICU admission and the 28 days in-hospital mortality rate.

Since the emergence of the COVID-19 pandemic, millions of people have become infected and died. (1-3). Despite the role of COVID-19 vaccines as a major tool in preventing the spread of disease, people are still at risk of infection and developing symptoms, even severe disease, and death (4,5). There are still many people in most low-income countries that have not gotten the vaccine yet or have not completed the vaccination course (6,7). Due to emergency use authorization for COVID-19 vaccines, the durability of the protection of COVID-19 vaccines is still unclear. Moreover, developing new variants of COVID-19 may impact the effectiveness of vaccines (8-10). As a result, it is assumed that first-generation COVID-19 vaccines may not completely bring the pandemic to an end (9). Therefore, effective treatment regimens for SARS-CoV-2 are crucially needed (3,11). Studies have demonstrated that Hydroxychloroquine, Lopinavir/Ritonavir, and Umifenovir showed little benefit in treating COVID-19 (12). Furthermore, Remdesivir and convalescence plasma may have promising benefits in cases with severe COVID-19 (13). In hospitalized patients with severe COVID-19, Tocilizumab has shown a reduced need for invasive mechanical ventilation and mortality (14). 

For COVID-19 patients treated by either mere oxygen or invasive mechanical ventilation, the use of dexamethasone led to reduced 28-day mortality but not for those treated with no respiratory support (15). Early treatment by Mulnipiravir, as the most recent oral antiviral therapy against SARS-CoV-2, has shown a decreased risk of hospitalization and mortality (16). Paxlovid, a combination of Nirmatrelvir and Ritonavir, has a course of treatment of 5 days which is given orally in patients with early COVID-19. It can reduce hospitalization and mortality in COVID-19 patients who are unvaccinated or with no COVID-19 history. The limited study population is one of the limitations of studies on Paxlovid. Also, Paxlovid is mostly evaluated in Omicron and Delta variants, leaving its efficacy on other variants and sub-lineages in question (17-19).

Based on molecular studies, SARS-CoV-2 inactivates angiotensin-converting enzyme 2 (ACE2), which leads to the accumulation of angiotensin II (Ang-II). Consequently, it increases inflammation and damages alveolar epithelium cells, leading to impaired gas exchange and respiratory failure (20). Determining the part that COVID-19 plays in respiratory failure requires unravelling a complicated network of vascular and inflammatory processes (21). We have speculated that the enhanced accessibility of Ang-II can be critical in infection pathophysiology. Because Ang-II signals function within the cell via the redox mechanism, we performed this study intending to block the intracellular function of Ang-II and prevent inflammation of alveolar cells and apoptosis (22). Moreover, hydrogen sulfide (H2S) is a protective agent against oxidative agents produced in humans (23). H2S also has a negative correlation with IL-6, a pro-inflammatory cytokine. This may lead to an additional anti-inflammatory effect of H2S. Patients with COVID-19 pneumonia develop low serum levels of H2S, which is a potential indicator of the severity of pneumonia (24). N-acetylcysteine (NAC) can increase H2S production by de-sulphuration to H2S and then oxidation to sulfane sulfur species that may act as an antioxidant agent (23). NAC is proven to be beneficial in variable contexts such as acute liver failure (25), acetaminophen poisoning (26), and lung diseases as a mucolytic anti-inflammatory (27). NAC, which is a mucolytic, provides cysteine, an essential precursor in glutathione (GSH) synthesis that can restore intracellular reduced GSH pools and therefore enhance the activity of the intracellular antioxidant system of glutathione pair (GSH-GSSG) (28). NAC is expected to function in several clinical states involving oxidating stress, such as ARDS, pneumonia, lung injuries, and bronchitis (29). Some clinical studies evaluated the effectiveness of NAC among respiratory diseases; one study showed a reduced need for ventilator support and better-off systemic oxygenation, but acute respiratory distress syndrome (ARDS) development and associated mortality did not improve significantly (30). While there have been many evaluations on the effectiveness of NAC in treating patients with viral lung infections, even during the pandemic of Influenza, there are still controversial results on the effectiveness of this thiol compound drug. 

In this article, we first aimed to evaluate and compare the clinical characteristics and laboratory findings at admission and on day 10 of the treatment among the NAC-received and control groups. Secondly, we intended to assess the hospitalization period and the upshot of the illness, e.g., the necessity of intubation, transfer to intensive care units (ICU), and 28-day in-hospital mortality. Third, we evaluated the clinical course and improvement of main COVID-19 symptoms and further made comparisons between the two groups.

## Methods

**Study design and participants: **This prospective case-control study has been aimed at assessing the safety and efficacy of intravenous NAC therapy's role on clinical outcomes, risk of ICU admission, and preventing respiratory failure within confirmed COVID-19 patients. This unicentric study has been carried out in the internal medicine department of Shahid Modarres Hospital, Shahid Beheshti University of Medical Sciences in Tehran, Iran, between June 11, 2020, and March 13, 2021. We prospectively enrolled the cases older than 40 with additional high-risk criteria of BMI ≥ 30 kg/m2, diabetes, and cardiovascular comorbidities, with a confirmed COVID-19 diagnosis using a real-time polymerase chain reaction test (rt-PCR) and the radiologic characteristics consistent with COVID-19 pneumonia. The exclusion criteria have been defined as follows: 1. Having known chronic respiratory disease (asthma, COPD), 2. Hypersensitivity and allergy to NAC, 3. Presence of the malignancy, 4. Pregnancy and having the lactating condition, 5. Signs of the impending requirement for orotracheal intubation (SaO2 < 90% with non-invasive oxygen therapy, reduced level of consciousness, increased respiratory effort), 6. Unstable hemodynamics, and 7. Reluctance to provide written consent. Finally, 217 patients were selected for the NAC group, and 245 patients were considered for the control group. 

**Dosage for NAC:** The experimental group included cases receiving a daily intravenous dosage of NAC at 1500 mg. Based on several studies on the potential beneficence of NAC on COVID-19 patients (including multiple clinical studies and extensive systematic reviews), it was suggested that the recommended optimum dosage of NAC from which COVID-19 patients could benefit was 1500 mg daily (31-34). NAC was diluted in 500cc of dextrose 5%, hence the 3 mg/cc concentration of each dosage. It was then administered via the IV route because some patients were NPO at different points of the study and could not take the drug orally. In fact, throughout the study, not all patients could consume medications via the enteral route. Therefore, we decided to have all the patients take NAC via the IV route to maintain homogeneity among all patients and to avoid any potential bias surrounding the different means of drug administration for different patients. Subsequently, the patients in the comparison arm were not administered NAC. The inclusion criteria for all control cases were the same as those for the cases in the NAC group. The evaluated variables have been recorded on the same datasheet, according to a standardized therapeutic method. 

**Case-control matching criteria & Randomization: **In this study, we used the stratified randomization method to make sure the potential influence of all covariates is accounted for among both study groups. Further, the cases in the control group have been matched with patients receiving NAC by SpO2/FiO2 ratio (within ±20) and age (within ±5 years). Based on the local protocols, all patients were administered oxygen support by a simple mask with a concentration of 10 liters per hour, ceftriaxone, azithromycin, vitamin D, ascorbic acid, and oseltamivir (tamiflu). The type and dosage of therapeutics were also matched between the groups. 

**Laboratorial and Radiological evaluation** **: **All enrolled patients in this study were confirmed by RT-PCR assays of nasopharyngeal specimens. Standard laboratory tests have been obtained from all patients, including white blood cells, lymphocytes, platelets, hemoglobin, C-reactive protein, and D-dimer. Chest computed tomography has been performed for all cases. The extent of lung involvement in the patient has been reported based on the classification protocol suggested by the Radiological Society of North America (RSNA) (35). Hence, we described the chest CT characteristics of our COVID-19 cases in four categories negative, atypical, intermediate, and typical.

**Statistical Analysis: **Categorical variables are recorded as percentages (%) and collated using chi-square or Fisher's exact test. Continuous data with a normal distribution are represented as the interquartile range (IQR) and median, then compared with the Mann-Whitney U-test. Statistical significance has been defined as a two-sided alpha lower than 0.05. SPSS Version 26.0 was used for all statistical evaluations.

**Sources of Bias: **Although we tried to nullify the unwanted influence of potential biases by using stratified randomization and case-control matching, due to the inherent nature of our study and patient selection, the methodology could still be prone to selection bias.

**Ethical consideration**: The study has been given approval by the Medical Ethical Committee of Shahid Beheshti University of Medical Sciences (ethical code: IR.SBMU.RETECH.REC.1399.049). The written informed consent has been acquired from all the participating cases, and if they could not grant consent, the respected legal representatives did so. All aspects of the study were conducted by protocols of the latest revision of the Helsinki declaration, 2008.

## Results

In this study, a total of 462 patients were enrolled, 217 cases were placed in the study group and 245 cases in the control group. The patients in the study group received NAC as adjuvant therapy, and the patients in the control group were treated conventionally without NAC. In the present research, we did all the necessary matching measures so the two groups would be similar in sociodemographic and clinical factors. Demonstrably, table 1 shows that cases in the NAC group and the control group have been statistically alike in terms of age, sex ratio, BMI, and smoking condition. Furthermore, table 1 indicates that from the medical history perspective, patients in the two groups were not significantly different from each other; the difference in the use of ACE inhibitors, Ang-II inhibitors, and statins between the two groups were all statistically insignificant. Also, it is shown that from the comorbidities point of view, the cases in the NAC group were considerably resembling the cases in the control group. Table 2 shows a comparison of clinical factors and lab results between the two groups at day-0 of treatment versus day-10 of treatment. The day-0 section of table 2 exhibits that at the start of the treatment process, there has not been a statistically significant difference among the clinical, laboratory, and radiological findings between the two groups (All the p-values in the day-0 section were greater than 0.05). After the treatment was started, two groups were once again evaluated at day-10 of treatment in terms of clinical, laboratory, and radiologic factors. The clinical findings, including forehead temperature, respiratory rate, and heart rate, showed no remarkable difference between the two groups. Among the laboratory findings, SpO2 level of the patients had a median of 91% in the NAC group and a median of 88% in the control group, marking a significant difference between the groups at day-10 of therapy with a p-value of 0.02. Furthermore, SpO2/FiO2, as another laboratory index, has been found to have a median of 463 in the NAC group, which was remarkably greater than 421, the median SpO2/FiO2 of the control group (P=0.01). Among the radiologic findings at day-10 of treatment, as reported by RSNA standards, no notable difference was recorded between the two groups.

Table 3 demonstrates the final clinical outcomes of patients in the NAC group versus those in the control group. The hospitalization period had a median of 19 days in the NAC group, significantly shorter than the median hospitalization period of 22 days in the control group (P=0.002). Other clinical outcomes including the need for ICU admission (P=0.31), ICU admission period (P=0.61), the need for intubation (p-value=0.11), and 28 days in-hospital mortality (P=0.37) yielded no statistically noteworthy difference between two groups. 

Figures 1A and 1B illustrate the clinical course of different important clinical factors for the NAC group and the control group, respectively. Fever and coughing as two of the common COVID-19 symptoms had the same time span between the two groups; fever starting on day 1 and ending on day 14 in both groups, and coughing starting on day 4 and ending on day 15 in both groups. Dyspnea, however, lasted one day more in the control group in comparison with the NAC group. ICU admission, as another important clinical determinant, lasted for seven days in both groups, but the NAC group required ICU admission one day later compared to the control group. The hospitalization period, as a key clinical outcome, was reported to be three days shorter for the NAC receiving cases than for the cases in the control group. The viral shedding, which was yet another determining factor of the patients' prognosis, lasted one day more for the patients of the NAC group as against the control group. Furthermore, ARDS and the need for mechanical ventilation happened two days and three day sooner in those who didn’t receive NAC, respectively. Lastly, death, as the ultimately adverse outcome, happened on day 18 for the cases of both groups. Figure 2 demonstrates the cumulative occurrence of different symptoms being compared between the two groups. Coughing (P=0.03), dyspnea (P=0.001), and loss of appetite (P=0.008) were all reported to have considerably lower cumulative occurrence in the NAC group. While other clinical manifestations including fever (P=0.7), fatigue (P=0.6), and myalgia (P=0.1) resulted in no significant difference between the two groups. 

**Table 1:** Sociodemographic information of hospitalized covid-19 patients divided into two groups receiving NAC adjuvant therapy and a control group (without NAC treatment)

**Parameter**	**NAC group**	**Control group**	**P-value**
**Total**	217	245	-
**Age**	62 (55, 71)	65 (53, 70)	0.13
**Gender (male)**	121 (55.7)	151 (61.6)	0.20
**BMI (kg/m2)**	33.4 (31.9, 36.7)	36 (31.1, 38.5)	0.28
**Smoker**			
**Yes**	19 (8.8)	26 (10.6)	0.50
**Pack-year**	8 (4, 9)	8 (5, 12)	0.10
**Previous use of ACE inhibitors**	16 (2.8)	23 (9.4)	0.43
**Previous use of Angiotensin 2 receptor blocker**	6 (2.7)	14 (5.7)	0.12
**Previous use of statin**	22 (10.1)	27 (11.0)	0.75
**History of recent contact with suspicious covid19 patient**	135 (62.2)	172 (70.2)	0.06
**Comorbidities**			
**Hypertension**	34 (15.7)	55 (22.4)	0.08
**Diabetes mellitus**	22 (10.1)	23 (9.4)	0.66
**Chronic liver disease**	4 (1.8)	6 (2.4)	0.65
**Chronic kidney disease**	15 (6.9)	20 (8.2)	0.61
**Cardiovascular disease**	133 (61.3)	141 (57.5)	0.41
**Days from symptom onset**	6 (5, 8)	6 (5, 11)	0.12

**Table 2:** The comparison of clinical and laboratory information of hospitalized covid-19 patients receiving NAC adjuvant therapy with the control group (without NAC treatment) in the day-0 (upon hospitalization) and Day-9

**Parameter**	**Day-0**			**Day-10**		
	**NAC-received group** **(N=217)**	**Control group** **(N=245)**	**p-value**	**NAC-received group** **(N=217)**	**Control group** **(N=245)**	**P-value**
**Clinical findings**						
**Forehead temperature**	37.9 (37.4, 38.4)	38.0 (37.2, 38.8)	0.48	36.4 (36.4, 36.9)	36.7 (36.3, 36.8)	0.20
**Respiratory rate per minute**	23 (19, 24)	23 (18, 25)	0.22	17 (16, 21)	18 (16, 22)	0.09
**Heart rate per minute**	88 (79, 93)	86 (80, 94)	0.39	80 (74, 86)	74 (70, 81)	0.11
**Laboratory findings**						
**SpO2% without oxygen support**	84 (79, 89)	85 (77, 91)	0.46	91 (87, 93)	88 (85, 91)	**0.02**
**SpO2% / FiO2**	261 (240, 257)	255 (241, 280)	0.77	463 (367, 459)	421 (321, 467)	**0.01**
**White blood cells (× 10** ^9^ **/L)**	6.1 (3.3, 8.1)	6.6 (4.5, 7.8)	0.71	7.4 (5.1, 10.1)	6.9 (6.2, 9.3)	0.59
**Lymphocytes (× 10** ^9^ **/L)**	0.7 (0.4, 0.9)	0.7 (0.4, 0.9)	0.57	1.9 (1.3, 1.6)	1.4 (0.9, 1.6)	0.63
**Platelets (× 10** ^9^ **/L)**	245 (103, 301)	194 (163, 294)	0.74	298 (193, 326)	221 (188; 301)	0.09
**Hemoglobin (gm/dl)**	12.5 (11.8, 13)	12.1 (11.9, 12.9)	0.41	12.7 (11.8, 13.3)	12.3 (12, 12.9)	0.21
**CRP (mg/L)**	94 (36, 101)	70 (53, 83)	0.15	8 (5, 15)	10 (7, 13)	0.07
**D dimer (ng/mL)**	1 (0.5, 1.1)	0.8 (0.6; 1.6)	0.09	0.8 (0.4, 1,0)	0.5 (0.3, 1.2)	0.19
**Radiological findings**						
**RSNA standard reporting of lung CT**						
**Typical**	179 (82.5)	211 (86.1)	0.28	183 (84.3)	217 (88.6)	0.18
**Intermediate**	35 (16.1)	25 (10.2)	0.06	32 (14.7)	23 (9.4)	0.07
**Atypical**	2 (0.9)	7 (2.8)	0.13	1 (0.5)	6 (2.4)	0.08
**Negative**	1 (0.5)	2 (0.8)	0.63	1 (0.5)	1 (0.4)	0.93
**Involvement in lung CT (%)**	52 (38,55)	38 (34,52)	0.25	36 (32, 38)	27 (32, 45)	0.08

**Table 3:** Clinical outcomes of the COVID-19 patients under treatment with adjuvant NAC compared to the control group (without treatment of NAC)

**Parameter**	**NAC-received (N=217)**	**Control group (N=245)**	**P-value**
**Days of Hospitalization**	19 (15, 21)	22 (17, 25)	**0.002**
**The Need for ICU Admission**	29 (13.3)	41 (16.7)	0.31
**Days of ICU Admission**	7 (5, 11)	7 (5, 9)	0.61
**Intubation or Invasive Mechanical Ventilation**	12 (5.5)	23 (9.4)	0.11
**28-day in-hospital Mortality**	10 (4.6)	16 (6.5)	0.37

**Figure 1A:**
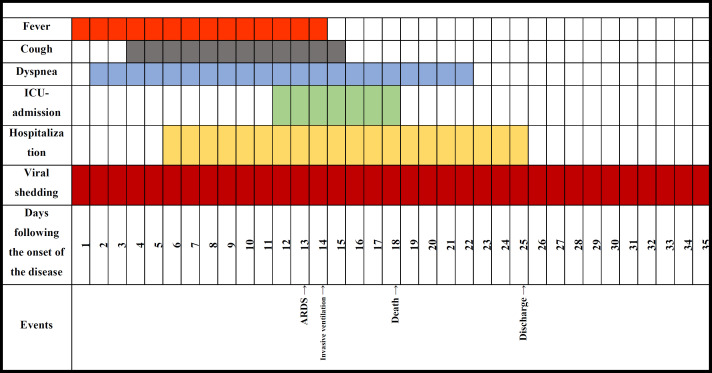
The clinical course of Hospitalized COVID-19 Patients Receiving NAC adjuvant Therapy

**Figure 1B:**
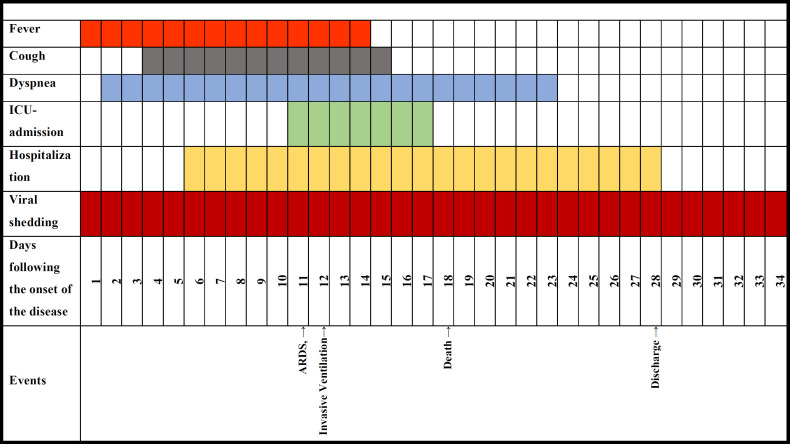
The clinical course of Hospitalized COVID-19 Patients without NAC adjuvant Therapy (Control Group)

**Figure 2:**
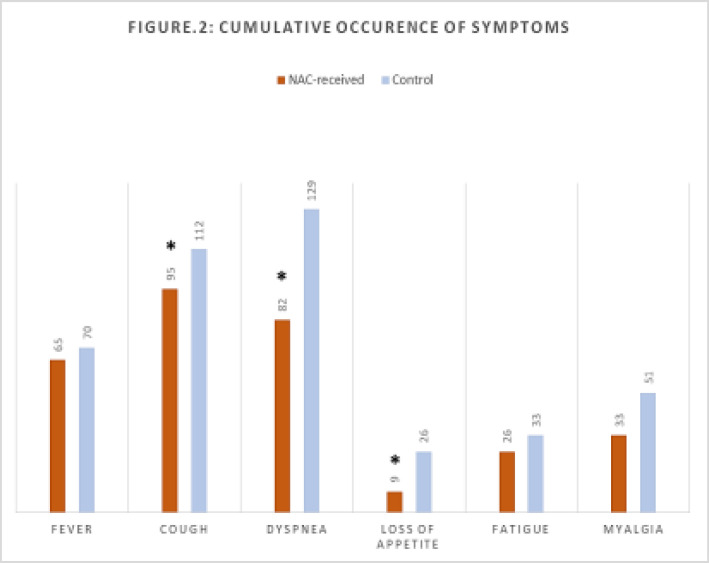
The cumulative occurrence of different symptoms in the COVID-19 patients treated with adjuvant N-Acetyl Cysteine (NAC) compared to the control group (without treatment of NAC)

*: p-value > 0.05. The comparison between the NAC group and control group was evaluated by Chi-squared analysis.

## Discussion

In this cohort study, we enrolled 462 hospitalized COVID-19 patients. The initial characteristics of the two groups were closely comparable in every way. Regarding the outcome, patients who received NAC had significantly shorter hospitalization periods, better SpO2% and SPO2/FiO2, and greater improvement in dyspnea, cough and loss of appetite compared to the control group. Notwithstanding, the NAC and control groups were not different in terms of the necessity of invasive mechanical ventilation, ICU admission, and 28 days in-hospital mortality rate.

 The two most recent published papers similarly achieved results regarding the role of NAC in the treatment strategy of SARS-CoV2 infected patients: The first study by N. Avdeev et al., which was performed on 46 hospitalized COVID-19 patients in Russia, reported that SpO2/FiO2 of patients was significantly enhanced after ten days of receiving NAC adjuvant therapy. Also, the patients receiving NAC had lower levels of C-reactive protein (CRP) as well as fewer days of hospitalization. They also reported The National Early Warning Score (NEWS2), as the scale of measuring physiological measurements routinely recorded at the patient's bedside, has significantly improved in NAC receiving group (36). The second study was conducted by J. Alencar et al. as a double-blinded case-placebo trial on 135 hospitalized cases in Brazil. The case group received 21 grams of NAC intravenously, separated into two dosages, 14gr in the initial four hours and 7gr in the next sixteen hours in one day. The findings did not support any significant difference in the need for ICU admission, intubation and mortality rate between NAC-received and placebo groups (20). Moreover, several articles have been published arguing that the redox mismatch induced by Ang-II can be a critical factor in the pathogenesis of SARS-CoV-2. (37-39).

Whilst the effects of NAC are found to be promising in mild or moderate patients in our study, the final prognosis and clinical outcomes of patients remained without significant change. Herein we should bear in mind the main possible reasons: Firstly, our study was mainly about the point of disease course at which NAC was administered. We excluded cases who had developed respiratory failure because of COVID-19 since we anticipated that redox signaling could not be used to reduce inflammation in these individuals (40). Each participant enrolled in the study exhibited signs of pulmonary infiltrations in their chest CT scans and required oxygen supplementations, indicating that they already had significant lung involvement. One could assume that prescribing NAC early on may be more beneficial at the symptoms' onset; nevertheless, it is known that the majority of these individuals will recover on their own. Needless to mention that it wouldn't be practical or morally acceptable to treat the individuals in a hospital for at least ten days, assuming that the vast majority of them will not require it. Secondly, we must not ignore the possibility that our original hypothesis may not be clinically working: excessive Ang-II has little effect on intracellular oxidative mismatch or disease etiology. It has been shown that there is a rise in Ang-II in the number of severe COVID-19 cases, although this might also be a coincidence and not have anything to do with the pathogenesis. Conversely, published articles demonstrated a better prognosis for patients using angiotensin receptor inhibitors. The evidence suggests that Ang-II might be somehow associated with SARS-CoV-2 pathogenesis (41). Moreover, it is notable that NAC was found to be successful in our study significantly decreasing some SARS-CoV2 infection symptoms, including local symptoms of the respiratory system such as coughing and general symptoms including loss of appetite and myalgia. The mentioned patterns indicate that the protective role of NAC is not only related to its mucolytic property but also possible to be attributed to other mechanisms containing antioxidant and immune-modulating mechanisms (2). 

We should keep in mind that the SARS-CoV2 virus infection - similar to other viral diseases, influenza, etc. is, in fact, related to clinical manifestations such as lack of appetite that suggests an involvement of tumor necrosis factor (TNF). The TNF production in phagocytes activated by viruses is boosted further by the accumulation of even minimal amounts of bacterial debris, such as lipopolysaccharides (42). This might clarify why severe consequences are so common in concurrent bacterial and influenza virus infections. These pathologic processes give justification for the therapeutic use of antioxidative agents in viral infections to restore the changed cell redox balance and avoid and/or cure clinical symptoms of immunological dysfunction. (43).

This study had some limitations required to be addressed: first, this study was unicentric. Second, the efficacy of NAC in combination with remdesivir and steroids as a robust drug was not evaluated. However, we must state that we did selectively administer a certain limited number of severe patients with steroids and remdesivir. If we wanted to include only these patients, the study population size would have been insufficiently small. And if we wanted to include these patients as an addition to others, the final results would have been highly affected by bias. As a matter of fact, if the patients receiving remdesivir and steroids were to be included in the study, the actual clinical effect of NAC might have been possibly masked by these two stronger medications, and the final results would have been biased. And thirdly, this study could be prone to selection bias due to the very nature of our methodology and patient selection, despite the fact that we attempted to nullify the bias as much as possible by proper matching criteria. On the other hand, the strength of this project was that we conducted the study prospectively, in contrast to most articles which evaluated the efficacy of NAC retrospectively.

 In fact, we have assessed the clinical course of patients, as well as important clinical endpoints such as 28-day fatality; on top of that, we have assessed NAC's pharmacological effect on the common symptoms, i.e., fever, fatigue, cough, and dyspnea. In conclusion, in this study, we showed that a daily intravenous dose of NAC at 1500 mg in hospitalized COVID-19 patients does not significantly change either the risk of ICU admission or the 28 days in-hospital mortality rate. However, the length of hospitalization and some patients' clinical symptoms may get better. This is while several papers persist in proposing focusing on Ang-II (44) or redox signals (45) as a viable therapeutic for COVID-19. The challenge is to intervene in the signaling cascades triggered by Ang-II without affecting individuals' renal function and hemodynamics. We propose that in the near term, the interaction between inflammation and Ang-II must be investigated more since they may provide fresh concepts for managing COVID-19. NAC is a safe, tolerable, available and affordable drug which seems like a promising adjuvant therapeutic agent for COVID-19. However, the therapeutic efficacy and optimal dose regimens of NAC must be studied further in bigger cohorts using randomized controlled clinical studies.
